# A Patient's Journey With Modern Melanoma Therapy

**DOI:** 10.7759/cureus.86811

**Published:** 2025-06-26

**Authors:** Alexander C Black, Gregory Senofsky

**Affiliations:** 1 Medicine, University of California Los Angeles David Geffen School of Medicine, Los Angeles, USA; 2 General Surgery, University of California Los Angeles, Los Angeles, USA

**Keywords:** adjuvant treatment, b raf mutation, immunotherapy, melanoma, sentinel lymph node dissection

## Abstract

This case report illustrates the current steps in the management of locally advanced melanoma treatment. This patient's journey involves modern surgical approaches to resectable melanoma and the indications for adjuvant therapy, and the use of immunotherapy and targeted therapy in advanced disease.

## Introduction

This patient was diagnosed with melanoma that was treated appropriately and then recurred, requiring subsequent and ongoing treatment. His journey reflects the current standard of care in melanoma therapy. On his initial presentation, he had standard wide excision and sentinel lymph node mapping and had Stage 2B melanoma [1]. He appropriately received no adjuvant therapy, which was not Federal Drug Administration (FDA) approved at the time. Upon loco-regional relapse, he received standard therapy first with doublet immunotherapy [2], which was stopped due to autoimmune toxicity, and then, given a B-raf mutation in his melanoma, doublet oral targeted therapy [3] from which he has had a sustained response.

## Case presentation

This patient had grown up in southern California with significant sun exposure, including time surfing, which resulted in a few blistering sun burns. Over several months, when he was 44 years old, he noted some itching and slight intermittent bleeding from a lower back mole. He was seen by dermatology for the irregularly margined and variable dark brown pigmented and symmetric lesion of 1.5 cm in maximum dimension and underwent a punch biopsy of a lesion in the midline lumbar back area, which revealed a superficial spreading melanoma with a significant vertical component, measuring 4.3 mm without ulceration (T4a). The pathology showed no regression or lymphovascular invasion, or satellitosis, and a low proliferative Ki-67 of 5%. The lesion was positive by immunohistochemical staining for Melan A and SOX 10, and p63. He subsequently underwent wide excision and sentinel lymph node resection, which remains the current standard of care for regional lymph node metastases assessment as summarized by Wong et al. [1]. The sentinel lymph node mapping led to bilateral groin lymph node sampling. His final pathology showed Stage 2B T4a N0 (no involved lymph nodes), and so he received no adjuvant therapy, since at the time, only Stage 3, or lymph node involved, melanoma received adjuvant therapy after surgery.

**Figure 1:**
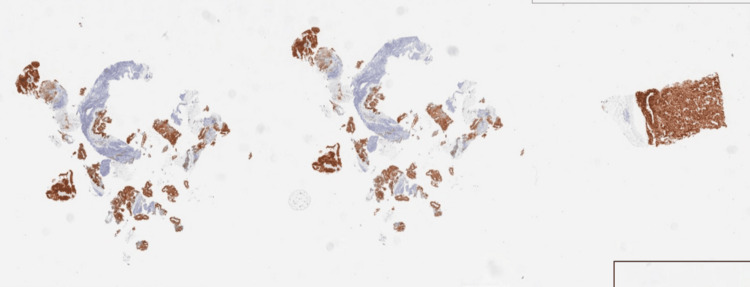
Right inguinal lymph node biopsy; November 2020 Brown stain for S-100

He remained free of melanoma for 3 ½ years but then developed right groin masses and subcutaneous lumps extending from the groin toward the buttock on the right. Figure 1 illustrates the lymph node biopsy confirming metastatic melanoma staining with S-100. He had molecular testing on the biopsy, which showed a B-raf V600E mutation.

His staging positron emission tomography (PET) scan showed no distant metastases. He was started on combination immunotherapy with nivolumab and ipilumumab, which targeted both PD-1/PD-L1 and CD28/ CTLA4 co-stimulatory immune regulatory pathways as was described by Larkin et al. [2]. He received two cycles but then developed unexplained fevers, fatigue, abnormal liver function tests, and thrombocytopenia. Given apparent immunotherapy toxicity, he had immunotherapy stopped and required prednisone 1 mg/kg orally daily on a tapering schedule for three months. Since his melanoma had a B-raf mutation, present in approximately 50% of melanomas, he was then given oral targeted therapy with dabrafenib 150 mg orally twice daily, which inhibited B-raf signaling, and trametinib 2 mg daily, which inhibited MEK signaling, as was reviewed by Robert et al. [3]. The addition of trametinib to dabrafenib minimized the stimulation of new skin squamous cell cancers, which were triggered by B-raf inhibition alone [3]. He had initial fatigue, diarrhea, and nausea, which subsided with continued therapy.

**Figure 2:**
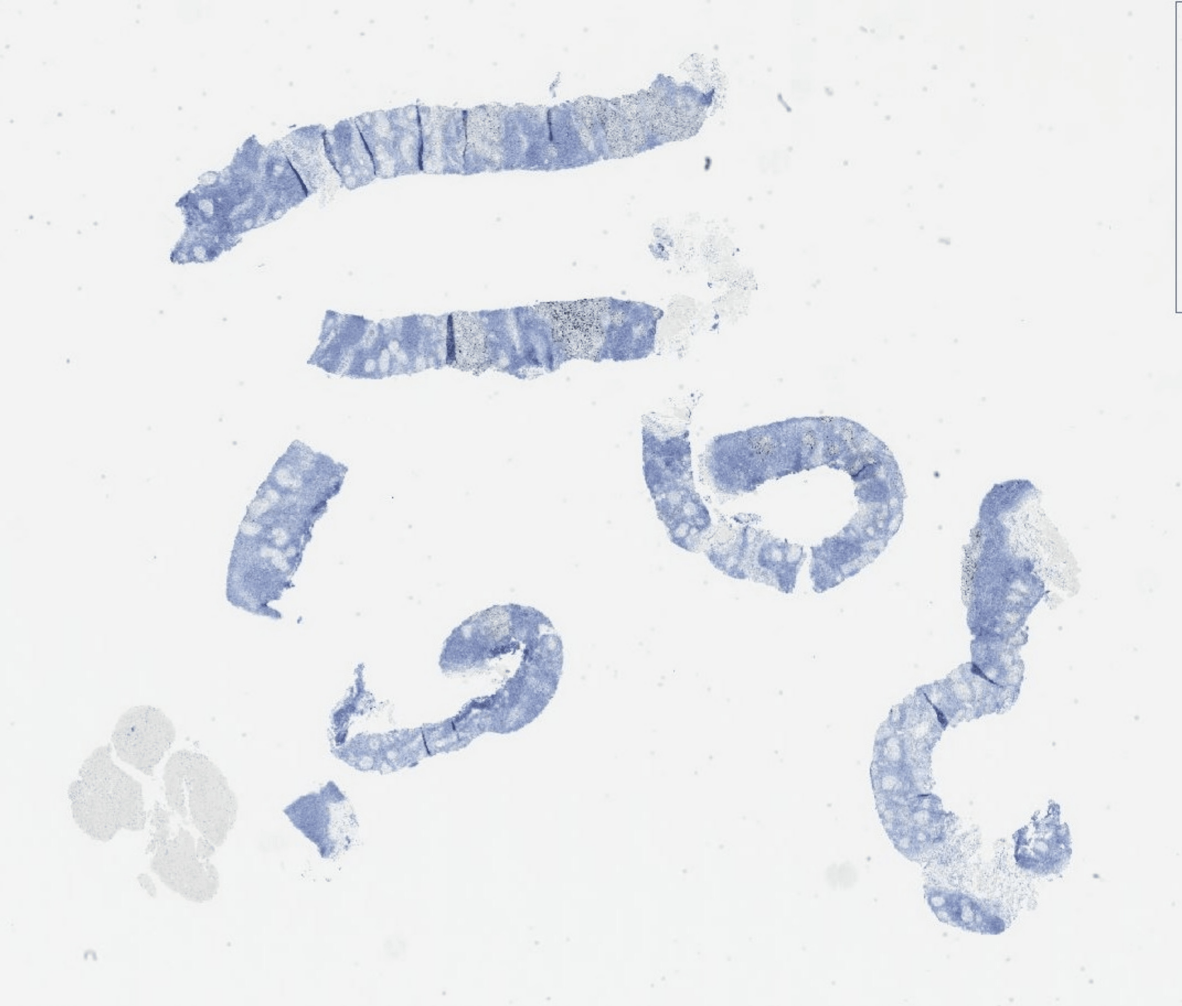
Right axillary lymph node biopsy; October 2023 Alcian blue stain illustrates the lighter granulomas in the lymph node

He had serial exams and intermittent repeat PET scans to confirm suppressed melanoma. There was no evidence of relapse until a PET 2 ½ years into dabrafenib and trametinib therapy revealed bilateral axillary adenopathy, which would have been less likely than a groin lymph node relapse. Figure 2 shows the pathology from an axillary lymph node biopsy, which revealed a non-caseating granuloma with negative acid-fast bacilli (AFB) and fungal tissue stains.

He had a mildly elevated angiotensin-converting enzyme (ACE) level of 100 U/L (normal 16-85) but no pulmonary imaging abnormalities or symptoms. His adenopathy gradually resolved, and he remains in a clinical and imaging remission over 3 ½ years into dual oral targeted therapy.

## Discussion

Systemic therapy has improved dramatically over the last 30 years. Initial use of chemotherapy resulted in a minority of patients having brief partial responses with no clear improvement in overall survival. Modest improvements in survival occurred with the use of older immune stimulatory approaches, including high-dose interleukin-2 (IL-2) infusions and alfa interferon (a IFN) intravenous and subcutaneous treatments at the price of substantial toxicity [4]. High-dose IL-2 created a septic shock-type reaction with hypotension, hypoxemia, and acute renal failure necessitating an intensive care unit (ICU) level of support while receiving the treatments. Alfa IFN was better tolerated but still caused flu-like symptoms and cytopenia, and liver inflammation.

This patient received double immunotherapy, which remains a first-line standard of care in metastatic melanoma, but which also can induce a myriad of autoimmune toxicities, resulting in stopping therapy with the patient after just two doses [2]. The small molecule targeted anti-B-raf dabrafenib induced high levels of response and markedly improved survival in metastatic melanoma, but induced a marked increase in cutaneous squamous cell cancers, felt to be due to reflex MEK activation to decreased B-raf signaling. This was blocked by adding the MEK inhibitor trametinib, allowing for sustained therapy and more durable responses [3]. Both immunotherapy and dual oral targeted therapy have also been shown to improve the cure rate in higher-risk non-metastatic melanoma, specifically Stage 2B and 3 for immunotherapy and Stage 3 for targeted therapy if a B-raf mutation is present, as reviewed by Luke et al. [5].

## Conclusions

This patient’s journey illustrates the benefits of understanding the molecular drivers of malignancy and harnessing the power of the body’s potential anti-tumor immune response. He received standard of care treatment throughout. He developed immunotherapy toxicity, which prompted early discontinuation, but fortunately, due to a targetable B-raf mutation, his melanoma has a sustained complete response to B-raf and MEK dual oral targeted therapy with minimal long-term side effects.
